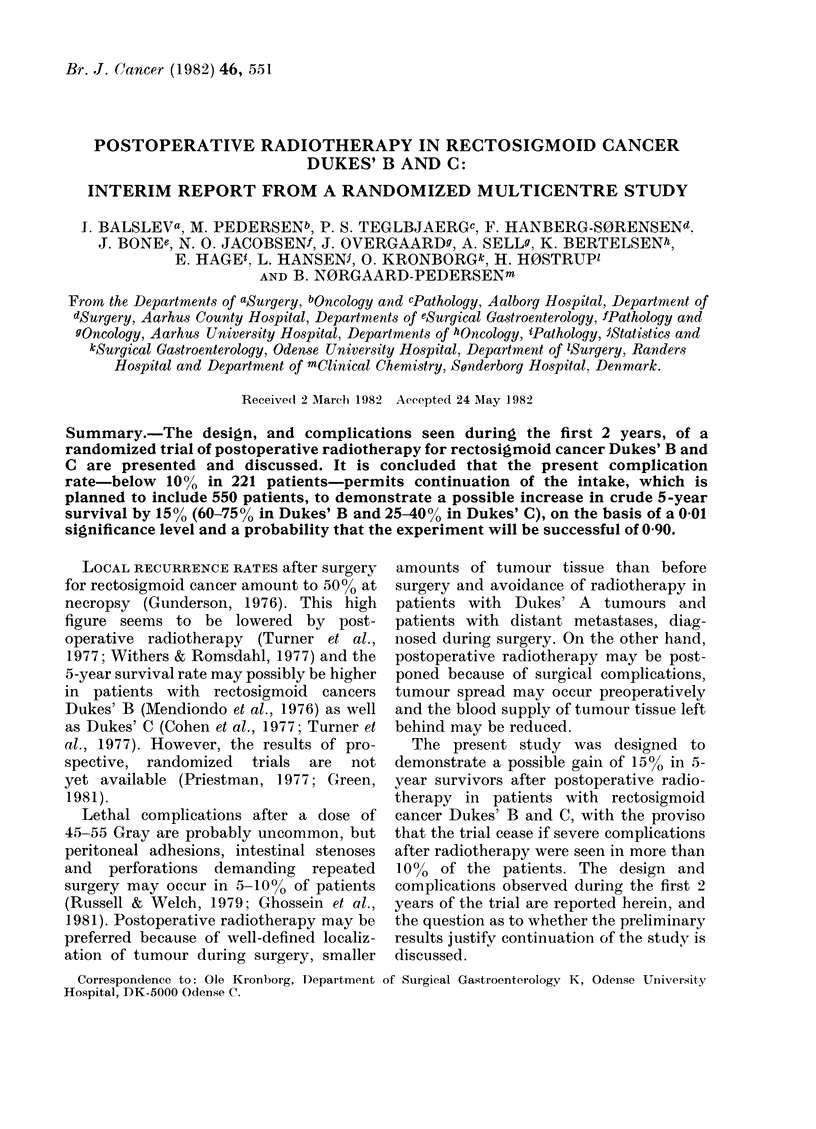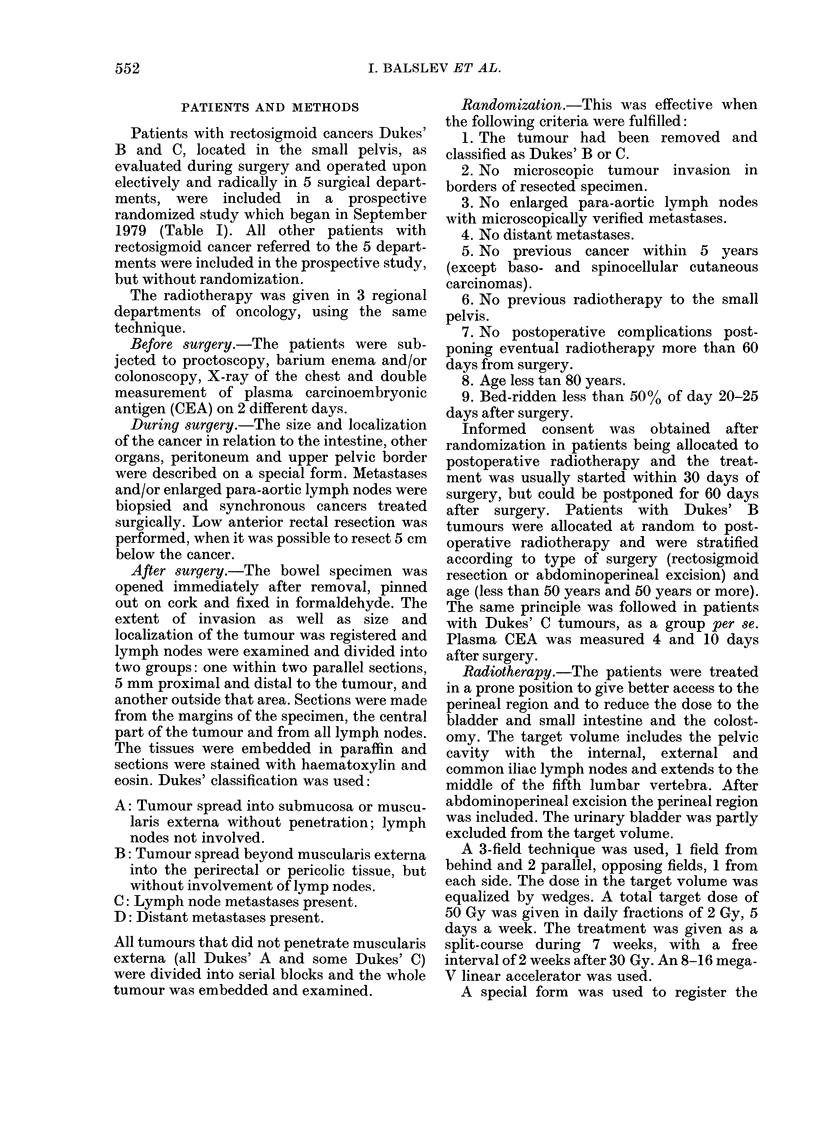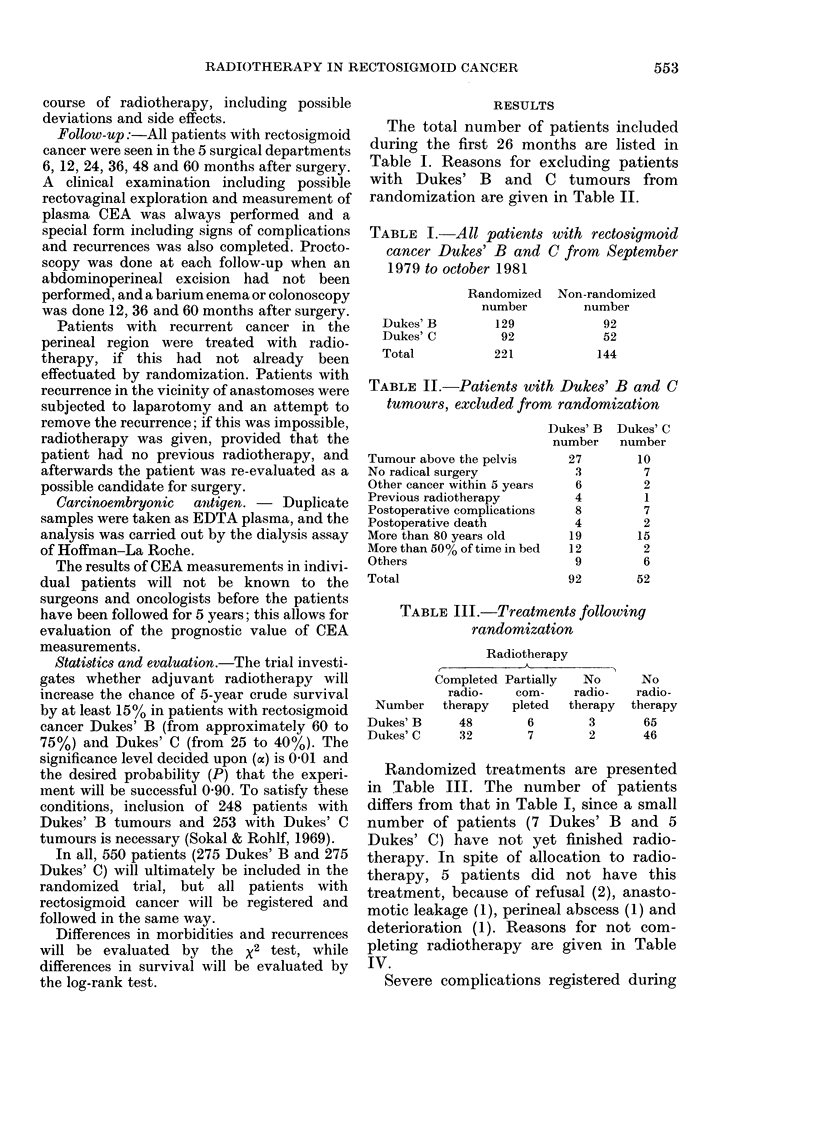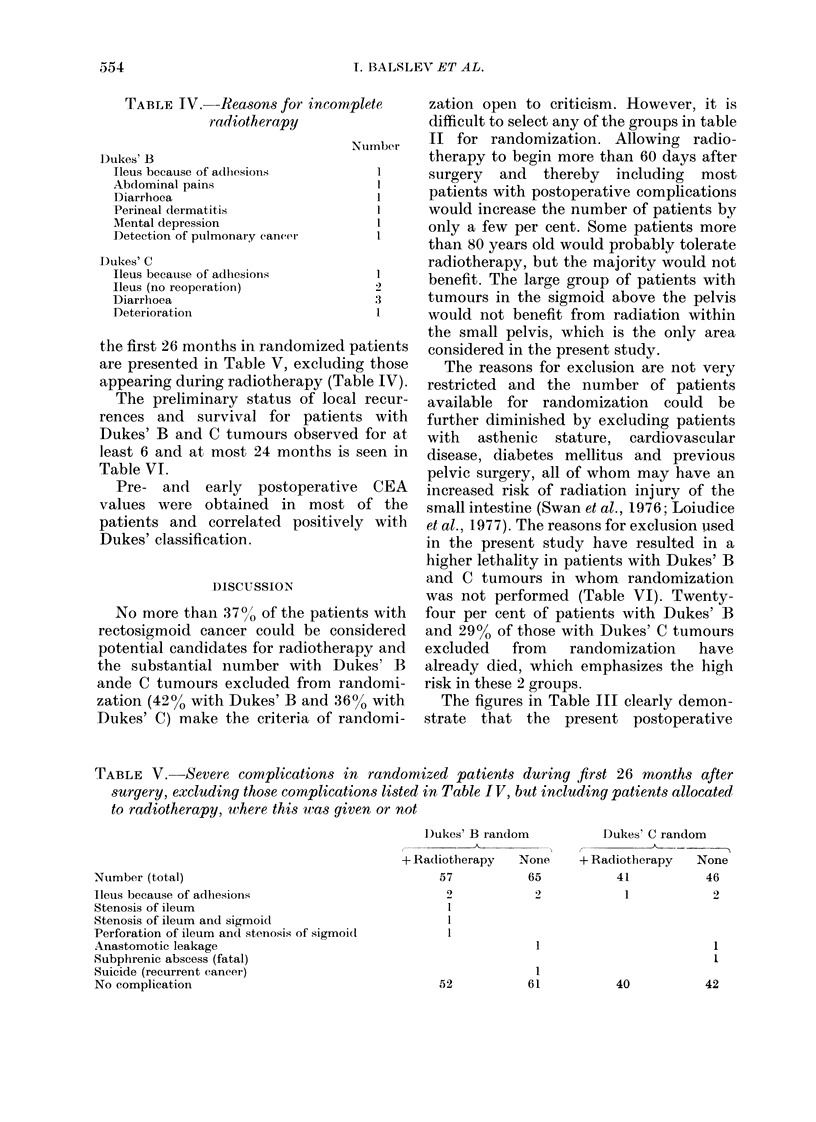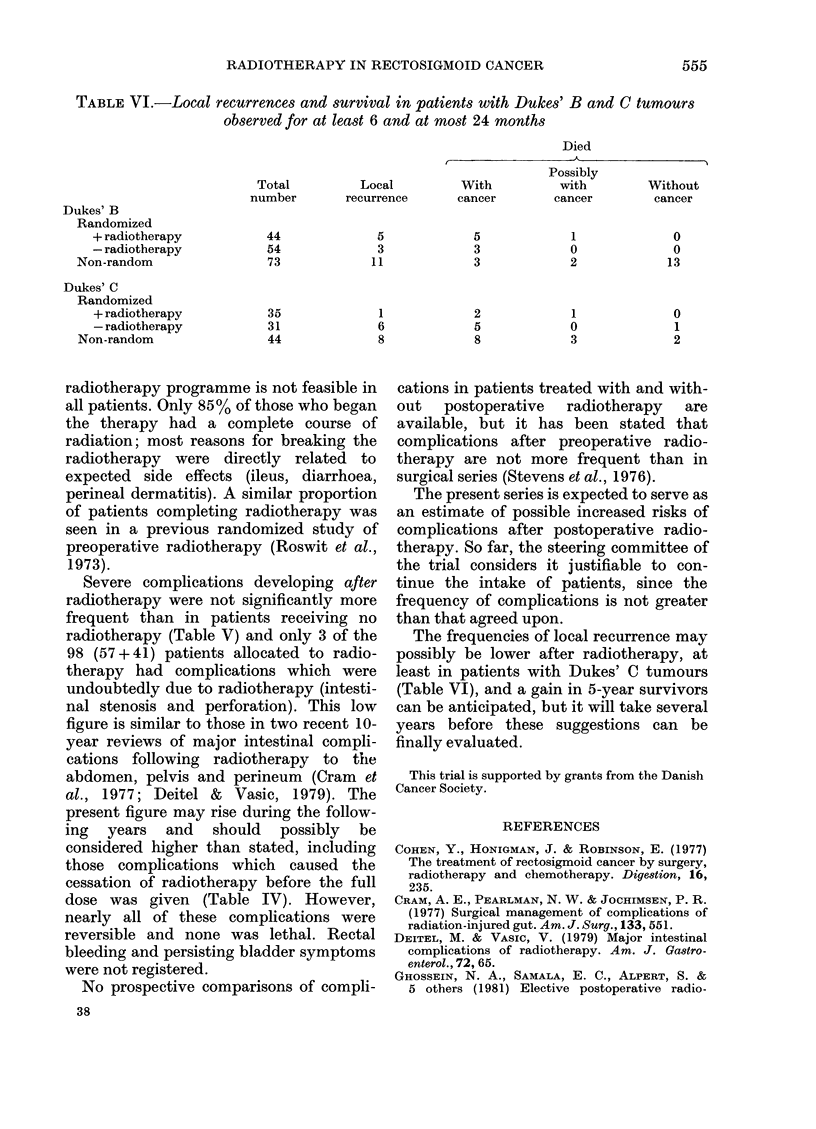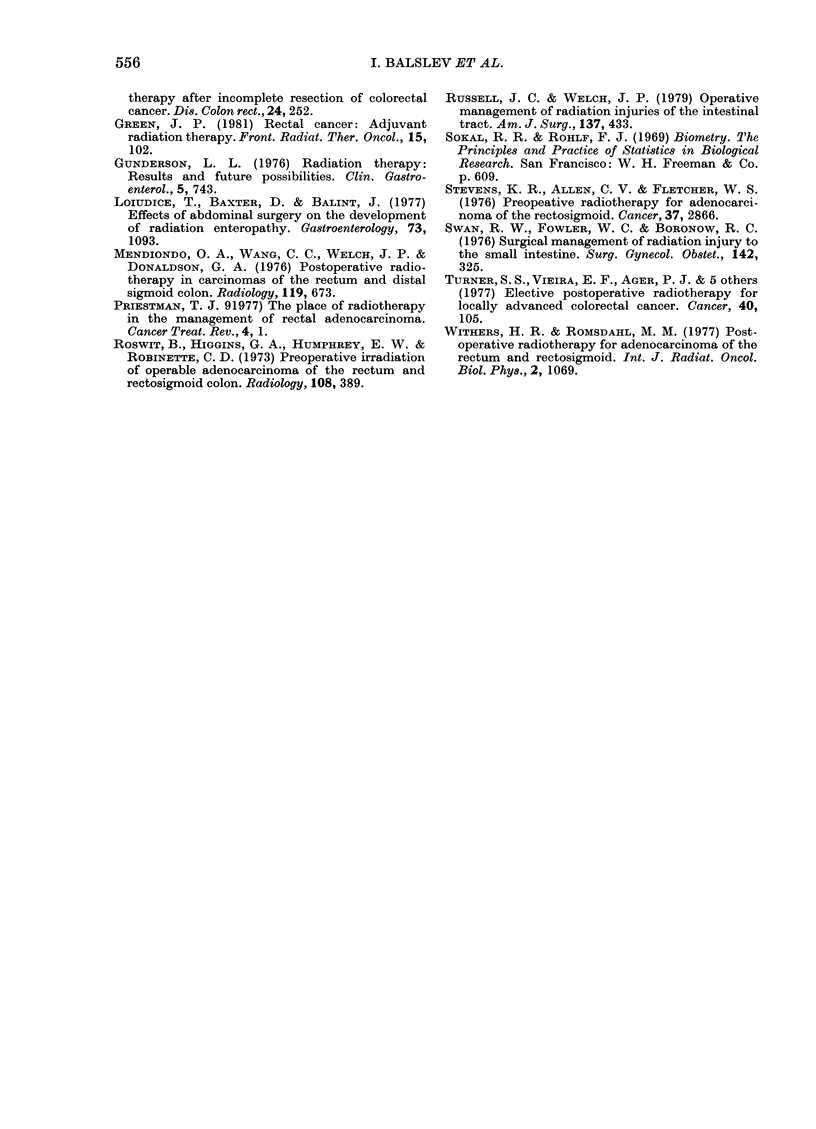# Postoperative radiotherapy in rectosigmoid cancer Dukes' B and C

**DOI:** 10.1038/bjc.1982.239

**Published:** 1982-10

**Authors:** I. Balslev, M. Pedersen, P. S. Teglbjaerg, F. Hanberg-Sørensen, J. Bone, N. O. Jacobsen, J. Overgaard, A. Sell, K. Bertelsen, E. Hage, L. Hansen, O. Kronborg, H. Høstrup, B. Nørgaard-Pedersen

## Abstract

The design, and complications seen during the first 2 years, of a randomized trial of postoperative radiotherapy for rectosigmoid cancer Dukes' B and C are presented and discussed. It is concluded that the present complication rate—below 10% in 221 patients—permits continuation of the intake, which is planned to include 550 patients, to demonstrate a possible increase in crude 5-year survival by 15% (60-75% in Dukes' B and 25-40% in Dukes' C), on the basis of a 0·01 significance level and a probability that the experiment will be successful of 0·90.


*Br. J. Cancer* (1982) **46**, 551

### POSTOPERATIVE RADIOTHERAPY IN RECTOSIGMOID CANCER DUKES' B AND C:

### INTERIM REPORT FROM A RANDOMIZED MULTICENTRE STUDY

I. BALSLEV[a], M. PEDERSEN[b], P. S. TEGLBJAERG[c], F. HANBERG-SØRENSEN[d], J. BONE[e], N. O. JACOBSEN[f], J. OVERGAARD[g], A. SELL[g], K. BERTELSEN[h], E. HAGE[i], L. HANSEN[j], O. KRONBORG[k], H. HØSTRUP[l] AND B. NØRGAARD-PEDERSEN[m]

*From the Departments of [a]Surgery, [b]Oncology and [c]Pathology, Aalborg Hospital, Department of [d]Surgery, Aarhus County Hospital, Departments of [e]Surgical Gastroenterology, [f]Pathology and [g]Oncology, Aarhus University Hospital, Departments of [h]Oncology, [i]Pathology, [j]Statistics and [k]Surgical Gastroenterology, Odense University Hospital, Department of [l]Surgery, Randers Hospital and Department of [m]Clinical Chemistry, Sønderborg Hospital, Denmark.*

Received 2 March 1982 Accepted 24 May 1982

**Summary.—The design, and complications seen during the first 2 years, of a randomized trial of postoperative radiotherapy for rectosigmoid cancer Dukes' B and C are presented and discussed. It is concluded that the present complication rate—below 10% in 221 patients—permits continuation of the intake, which is planned to include 550 patients, to demonstrate a possible increase in crude 5-year survival by 15% (60–75% in Dukes' B and 25–40% in Dukes' C), on the basis of a 0·01 significance level and a probability that the experiment will be successful of 0·90.**

LOCAL RECURRENCE RATES after surgery for rectosigmoid cancer amount to 50% at necropsy (Gunderson, 1976). This high figure seems to be lowered by postoperative radiotherapy (Turner *et al.*, 1977; Withers & Romsdahl, 1977) and the 5-year survival rate may possibly be higher in patients with rectosigmoid cancers Dukes' B (Mendiondo *et al.*, 1976) as well as Dukes' C (Cohen *et al.*, 1977; Turner *et al.*, 1977). However, the results of prospective, randomized trials are not yet available (Priestman, 1977; Green, 1981).

Lethal complications after a dose of 45–55 Gray are probably uncommon, but peritoneal adhesions, intestinal stenoses and perforations demanding repeated surgery may occur in 5–10% of patients (Russell & Welch, 1979; Ghossein *et al.*, 1981). Postoperative radiotherapy may be preferred because of well-defined localization of tumour during surgery, smaller amounts of tumour tissue than before surgery and avoidance of radiotherapy in patients with Dukes' A tumours and patients with distant metastases, diagnosed during surgery. On the other hand, postoperative radiotherapy may be postponed because of surgical complications, tumour spread may occur preoperatively and the blood supply of tumour tissue left behind may be reduced.

The present study was designed to demonstrate a possible gain of 15% in 5-year survivors after postoperative radiotherapy in patients with rectosigmoid cancer Dukes' B and C, with the proviso that the trial cease if severe complications after radiotherapy were seen in more than 10% of the patients. The design and complications observed during the first 2 years of the trial are reported herein, and the question as to whether the preliminary results justify continuation of the study is discussed.

Correspondence to: Ole Kronborg, Department of Surgical Gastroenterology K, Odense University Hospital, DK-5000 Odense C.

### PATIENTS AND METHODS

Patients with rectosigmoid cancers Dukes' B and C, located in the small pelvis, as evaluated during surgery and operated upon electively and radically in 5 surgical departments, were included in a prospective randomized study which began in September 1979 (Table I). All other patients with rectosigmoid cancer referred to the 5 departments were included in the prospective study, but without randomization.

The radiotherapy was given in 3 regional departments of oncology, using the same technique.

*Before surgery.*—The patients were subjected to proctoscopy, barium enema and/or colonoscopy, X-ray of the chest and double measurement of plasma carcinoembryonic antigen (CEA) on 2 different days.

*During surgery.*—The size and localization of the cancer in relation to the intestine, other organs, peritoneum and upper pelvic border were described on a special form. Metastases and/or enlarged para-aortic lymph nodes were biopsied and synchronous cancers treated surgically. Low anterior rectal resection was performed, when it was possible to resect 5 cm below the cancer.

*After surgery.*—The bowel specimen was opened immediately after removal, pinned out on cork and fixed in formaldehyde. The extent of invasion as well as size and localization of the tumour was registered and lymph nodes were examined and divided into two groups: one within two parallel sections, 5 mm proximal and distal to the tumour, and another outside that area. Sections were made from the margins of the specimen, the central part of the tumour and from all lymph nodes. The tissues were embedded in paraffin and sections were stained with haematoxylin and eosin. Dukes' classification was used:

A: Tumour spread into submucosa or muscularis externa without penetration; lymph nodes not involved.
B: Tumour spread beyond muscularis externa into the perirectal or pericolic tissue, but without involvement of lymp nodes.
C: Lymph node metastases present.
D: Distant metastases present.

All tumours that did not penetrate muscularis externa (all Dukes' A and some Dukes' C) were divided into serial blocks and the whole tumour was embedded and examined.

*Randomization.*—This was effective when the following criteria were fulfilled:

1. The tumour had been removed and classified as Dukes' B or C.
2. No microscopic tumour invasion in borders of resected specimen.
3. No enlarged para-aortic lymph nodes with microscopically verified metastases.
4. No distant metastases.
5. No previous cancer within 5 years (except baso- and spinocellular cutaneous carcinomas).
6. No previous radiotherapy to the small pelvis.
7. No postoperative complications postponing eventual radiotherapy more than 60 days from surgery.
8. Age less tan 80 years.
9. Bed-ridden less than 50% of day 20–25 days after surgery.

Informed consent was obtained after randomization in patients being allocated to postoperative radiotherapy and the treatment was usually started within 30 days of surgery, but could be postponed for 60 days after surgery. Patients with Dukes' B tumours were allocated at random to postoperative radiotherapy and were stratified according to type of surgery (rectosigmoid resection or abdominoperineal excision) and age (less than 50 years and 50 years or more). The same principle was followed in patients with Dukes' C tumours, as a group *per se*. Plasma CEA was measured 4 and 10 days after surgery.

*Radiotherapy.*—The patients were treated in a prone position to give better access to the perineal region and to reduce the dose to the bladder and small intestine and the colostomy. The target volume includes the pelvic cavity with the internal, external and common iliac lymph nodes and extends to the middle of the fifth lumbar vertebra. After abdominoperineal excision the perineal region was included. The urinary bladder was partly excluded from the target volume.

A 3-field technique was used, 1 field from behind and 2 parallel, opposing fields, 1 from each side. The dose in the target volume was equalized by wedges. A total target dose of 50 Gy was given in daily fractions of 2 Gy, 5 days a week. The treatment was given as a split-course during 7 weeks, with a free interval of 2 weeks after 30 Gy. An 8–16 mega-V linear accelerator was used.

A special form was used to register the course of radiotherapy, including possible deviations and side effects.

*Follow-up:*—All patients with rectosigmoid cancer were seen in the 5 surgical departments 6, 12, 24, 36, 48 and 60 months after surgery. A clinical examination including possible rectovaginal exploration and measurement of plasma CEA was always performed and a special form including signs of complications and recurrences was also completed. Proctoscopy was done at each follow-up when an abdominoperineal excision had not been performed, and a barium enema or colonoscopy was done 12, 36 and 60 months after surgery.

Patients with recurrent cancer in the perineal region were treated with radiotherapy, if this had not already been effectuated by randomization. Patients with recurrence in the vicinity of anastomoses were subjected to laparotomy and an attempt to remove the recurrence; if this was impossible, radiotherapy was given, provided that the patient had no previous radiotherapy, and afterwards the patient was re-evaluated as a possible candidate for surgery.

*Carcinoembryonic antigen.* — Duplicate samples were taken as EDTA plasma, and the analysis was carried out by the dialysis assay of Hoffman–La Roche.

The results of CEA measurements in individual patients will not be known to the surgeons and oncologists before the patients have been followed for 5 years; this allows for evaluation of the prognostic value of CEA measurements.

*Statistics and evaluation.*—The trial investigates whether adjuvant radiotherapy will increase the chance of 5-year crude survival by at least 15% in patients with rectosigmoid cancer Dukes' B (from approximately 60 to 75%) and Dukes' C (from 25 to 40%). The significance level decided upon ($\alpha$) is 0·01 and the desired probability ($P$) that the experiment will be successful 0·90. To satisfy these conditions, inclusion of 248 patients with Dukes' B tumours and 253 with Dukes' C tumours is necessary (Sokal & Rohlf, 1969).

In all, 550 patients (275 Dukes' B and 275 Dukes' C) will ultimately be included in the randomized trial, but all patients with rectosigmoid cancer will be registered and followed in the same way.

Differences in morbidities and recurrences will be evaluated by the $\chi^2$ test, while differences in survival will be evaluated by the log-rank test.

### RESULTS

The total number of patients included during the first 26 months are listed in Table I. Reasons for excluding patients with Dukes' B and C tumours from randomization are given in Table II.

TABLE I.—*All patients with rectosigmoid cancer Dukes' B and C from September* 1979 *to october* 1981

| | Randomized number | Non-randomized number |
|---|---|---|
| Dukes' B | 129 | 92 |
| Dukes' C | 92 | 52 |
| Total | 221 | 144 |

TABLE II.—*Patients with Dukes' B and C tumours, excluded from randomization*

| | Dukes' B number | Dukes' C number |
|---|---|---|
| Tumour above the pelvis | 27 | 10 |
| No radical surgery | 3 | 7 |
| Other cancer within 5 years | 6 | 2 |
| Previous radiotherapy | 4 | 1 |
| Postoperative complications | 8 | 7 |
| Postoperative death | 4 | 2 |
| More than 80 years old | 19 | 15 |
| More than 50% of time in bed | 12 | 2 |
| Others | 9 | 6 |
| Total | 92 | 52 |

TABLE III.—*Treatments following randomization*

| | Radiotherapy | | | |
|---|---|---|---|---|
| Number | Completed radiotherapy | Partially completed | No radiotherapy | No radiotherapy |
| Dukes' B | 48 | 6 | 3 | 65 |
| Dukes' C | 32 | 7 | 2 | 46 |

Randomized treatments are presented in Table III. The number of patients differs from that in Table I, since a small number of patients (7 Dukes' B and 5 Dukes' C) have not yet finished radiotherapy. In spite of allocation to radiotherapy, 5 patients did not have this treatment, because of refusal (2), anastomotic leakage (1), perineal abscess (1) and deterioration (1). Reasons for not completing radiotherapy are given in Table IV.

Severe complications registered during

TABLE IV.—*Reasons for incomplete radiotherapy*

| | Number |
|---|---|
| Dukes' B | |
| Ileus because of adhesions | 1 |
| Abdominal pains | 1 |
| Diarrhoea | 1 |
| Perineal dermatitis | 1 |
| Mental depression | 1 |
| Detection of pulmonary cancer | 1 |
| Dukes' C | |
| Ileus because of adhesions | 1 |
| Ileus (no reoperation) | 2 |
| Diarrhoea | 3 |
| Deterioration | 1 |

the first 26 months in randomized patients are presented in Table V, excluding those appearing during radiotherapy (Table IV).

The preliminary status of local recurrences and survival for patients with Dukes' B and C tumours observed for at least 6 and at most 24 months is seen in Table VI.

Pre- and early postoperative CEA values were obtained in most of the patients and correlated positively with Dukes' classification.

### DISCUSSION

No more than 37% of the patients with rectosigmoid cancer could be considered potential candidates for radiotherapy and the substantial number with Dukes' B ande C tumours excluded from randomization (42% with Dukes' B and 36% with Dukes' C) make the criteria of randomization open to criticism. However, it is difficult to select any of the groups in table II for randomization. Allowing radiotherapy to begin more than 60 days after surgery and thereby including most patients with postoperative complications would increase the number of patients by only a few per cent. Some patients more than 80 years old would probably tolerate radiotherapy, but the majority would not benefit. The large group of patients with tumours in the sigmoid above the pelvis would not benefit from radiation within the small pelvis, which is the only area considered in the present study.

The reasons for exclusion are not very restricted and the number of patients available for randomization could be further diminished by excluding patients with asthenic stature, cardiovascular disease, diabetes mellitus and previous pelvic surgery, all of whom may have an increased risk of radiation injury of the small intestine (Swan *et al.*, 1976; Loiudice *et al.*, 1977). The reasons for exclusion used in the present study have resulted in a higher lethality in patients with Dukes' B and C tumours in whom randomization was not performed (Table VI). Twenty-four per cent of patients with Dukes' B and 29% of those with Dukes' C tumours excluded from randomization have already died, which emphasizes the high risk in these 2 groups.

The figures in Table III clearly demonstrate that the present postoperative

TABLE V.—*Severe complications in randomized patients during first 26 months after surgery, excluding those complications listed in Table IV, but including patients allocated to radiotherapy, where this was given or not*

| | Dukes' B random | | Dukes' C random | |
|---|---|---|---|---|
| | +Radiotherapy | None | +Radiotherapy | None |
| Number (total) | 57 | 65 | 41 | 46 |
| Ileus because of adhesions | 2 | 2 | 1 | 2 |
| Stenosis of ileum | 1 | | | |
| Stenosis of ileum and sigmoid | 1 | | | |
| Perforation of ileum and stenosis of sigmoid | 1 | | | |
| Anastomotic leakage | | 1 | | 1 |
| Subphrenic abscess (fatal) | | | | 1 |
| Suicide (recurrent cancer) | | 1 | | |
| No complication | 52 | 61 | 40 | 42 |

TABLE VI.—*Local recurrences and survival in patients with Dukes' B and C tumours observed for at least 6 and at most 24 months*

| | Total number | Local recurrence | Died: With cancer | Died: Possibly with cancer | Died: Without cancer |
|---|---|---|---|---|---|
| Dukes' B | | | | | |
| Randomized | | | | | |
| + radiotherapy | 44 | 5 | 5 | 1 | 0 |
| − radiotherapy | 54 | 3 | 3 | 0 | 0 |
| Non-random | 73 | 11 | 3 | 2 | 13 |
| Dukes' C | | | | | |
| Randomized | | | | | |
| + radiotherapy | 35 | 1 | 2 | 1 | 0 |
| − radiotherapy | 31 | 6 | 5 | 0 | 1 |
| Non-random | 44 | 8 | 8 | 3 | 2 |

radiotherapy programme is not feasible in all patients. Only 85% of those who began the therapy had a complete course of radiation; most reasons for breaking the radiotherapy were directly related to expected side effects (ileus, diarrhoea, perineal dermatitis). A similar proportion of patients completing radiotherapy was seen in a previous randomized study of preoperative radiotherapy (Roswit *et al.*, 1973).

Severe complications developing *after* radiotherapy were not significantly more frequent than in patients receiving no radiotherapy (Table V) and only 3 of the 98 (57 + 41) patients allocated to radiotherapy had complications which were undoubtedly due to radiotherapy (intestinal stenosis and perforation). This low figure is similar to those in two recent 10-year reviews of major intestinal complications following radiotherapy to the abdomen, pelvis and perineum (Cram *et al.*, 1977; Deitel & Vasic, 1979). The present figure may rise during the following years and should possibly be considered higher than stated, including those complications which caused the cessation of radiotherapy before the full dose was given (Table IV). However, nearly all of these complications were reversible and none was lethal. Rectal bleeding and persisting bladder symptoms were not registered.

No prospective comparisons of complications in patients treated with and without postoperative radiotherapy are available, but it has been stated that complications after preoperative radiotherapy are not more frequent than in surgical series (Stevens *et al.*, 1976).

The present series is expected to serve as an estimate of possible increased risks of complications after postoperative radiotherapy. So far, the steering committee of the trial considers it justifiable to continue the intake of patients, since the frequency of complications is not greater than that agreed upon.

The frequencies of local recurrence may possibly be lower after radiotherapy, at least in patients with Dukes' C tumours (Table VI), and a gain in 5-year survivors can be anticipated, but it will take several years before these suggestions can be finally evaluated.

This trial is supported by grants from the Danish Cancer Society.

REFERENCES

COHEN, Y., HONIGMAN, J. & ROBINSON, E. (1977) The treatment of rectosigmoid cancer by surgery, radiotherapy and chemotherapy. *Digestion*, **16**, 235.

CRAM, A. E., PEARLMAN, N. W. & JOCHIMSEN, P. R. (1977) Surgical management of complications of radiation-injured gut. *Am. J. Surg.*, **133**, 551.

DEITEL, M. & VASIC, V. (1979) Major intestinal complications of radiotherapy. *Am. J. Gastroenterol.*, **72**, 65.

GHOSSEIN, N. A., SAMALA, E. C., ALPERT, S. & 5 others (1981) Elective postoperative radiotherapy after incomplete resection of colorectal cancer. *Dis. Colon rect.*, **24**, 252.

GREEN, J. P. (1981) Rectal cancer: Adjuvant radiation therapy. *Front. Radiat. Ther. Oncol.*, **15**, 102.

GUNDERSON, L. L. (1976) Radiation therapy: Results and future possibilities. *Clin. Gastroenterol.*, **5**, 743.

LOIUDICE, T., BAXTER, D. & BALINT, J. (1977) Effects of abdominal surgery on the development of radiation enteropathy. *Gastroenterology*, **73**, 1093.

MENDIONDO, O. A., WANG, C. C., WELCH, J. P. & DONALDSON, G. A. (1976) Postoperative radiotherapy in carcinomas of the rectum and distal sigmoid colon. *Radiology*, **119**, 673.

PRIESTMAN, T. J. 91977) The place of radiotherapy in the management of rectal adenocarcinoma. *Cancer Treat. Rev.*, **4**, 1.

ROSWIT, B., HIGGINS, G. A., HUMPHREY, E. W. & ROBINETTE, C. D. (1973) Preoperative irradiation of operable adenocarcinoma of the rectum and rectosigmoid colon. *Radiology*, **108**, 389.

RUSSELL, J. C. & WELCH, J. P. (1979) Operative management of radiation injuries of the intestinal tract. *Am. J. Surg.*, **137**, 433.

SOKAL, R. R. & ROHLF, F. J. (1969) *Biometry. The Principles and Practice of Statistics in Biological Research.* San Francisco: W. H. Freeman & Co. p. 609.

STEVENS, K. R., ALLEN, C. V. & FLETCHER, W. S. (1976) Preopeative radiotherapy for adenocarcinoma of the rectosigmoid. *Cancer*, **37**, 2866.

SWAN, R. W., FOWLER, W. C. & BORONOW, R. C. (1976) Surgical management of radiation injury to the small intestine. *Surg. Gynecol. Obstet.*, **142**, 325.

TURNER, S. S., VIEIRA, E. F., AGER, P. J. & 5 others (1977) Elective postoperative radiotherapy for locally advanced colorectal cancer. *Cancer*, **40**, 105.

WITHERS, H. R. & ROMSDAHL, M. M. (1977) Postoperative radiotherapy for adenocarcinoma of the rectum and rectosigmoid. *Int. J. Radiat. Oncol. Biol. Phys.*, **2**, 1069.